# From Screen to Plate: How Instagram Cooking Videos Promote Healthy Eating Behaviours in Established Adulthood

**DOI:** 10.3390/nu17071133

**Published:** 2025-03-25

**Authors:** Yen-Cheng Chen, Ching-Sung Lee, Ming-Chen Chiang, Pei-Ling Tsui

**Affiliations:** 1 Department of Applied Science of Living, Chinese Culture University, Taipei 11114, Taiwan; cyc4@ulive.pccu.edu.tw; 2 Department of Restaurant, Hotel and Institutional Management, Fu Jen Catholic University, New Taipei City 242062, Taiwan; 3 Ph. D. Program in Nutrition and Food Science, College of Human Ecology, Fu Jen Catholic University, New Taipei City 242062, Taiwan; 4 Department of Hospitality Management, National Taitung Junior College, Taitung 95045, Taiwan; 5 Graduate Institute of Technological and Vocational Education, National Taipei University of Technology, Taipei 10608, Taiwan

**Keywords:** social media, healthy eating behaviours, cooking videos, Instagram, nutrition promotion, established adulthood, public health, dietary behaviour

## Abstract

Background: Social media platforms increasingly influence dietary behaviours, with cooking videos emerging as a key tool for promoting healthy eating. However, limited research has examined how such digital content affects adults in established adulthood. Objective: This study investigates the relationships among cooking behaviour, engagement with healthy cooking videos on Instagram, and healthy eating behaviours among Taiwanese adults aged 30 to 45. Methods: A cross-sectional online survey collected valid responses from 488 participants (effective response rate = 81.3%) who regularly cook and engage with food-related content on Instagram. Structural equation modelling (SEM) was used to test hypothesised relationships. Results: Cooking behaviour was positively associated with engagement in healthy cooking multimedia (β = 0.262, *p* < 0.01). Engagement significantly predicted healthy eating behaviours (β = 0.399, *p* < 0.001) and mediated the effect of cooking behaviour on dietary outcomes (indirect effect = 0.105, 95% CI [0.044, 0.204]). Cooking behaviour alone was not directly associated with healthy eating behaviours (β = 0.009, n.s.). Conclusions: Engagement with healthy cooking videos enhances healthy eating practices among adults in established adulthood. These findings highlight Instagram’s potential as a digital health promotion tool and support the integration of culinary media into public nutrition strategies.

## 1. Introduction

Social media has become a dominant global force in contemporary culture [1]. The rapid expansion of these platforms has further transformed cooking into an interactive and visually engaging experience, increasing accessibility and motivation for individuals to participate in culinary activities [2,3]. Cooking videos are a prominent feature across various social media platforms, serving as one of the many mediums through which food content is presented to audiences [4]. In particular, cooking videos on Instagram have garnered widespread attention due to their rich visual and auditory elements. These features not only enhance viewers’ understanding of cooking techniques but also serve as motivational tools that encourage active participation [2,3]. Previous studies suggest that such videos facilitate virtual engagement, potentially increasing individuals’ motivation and confidence in cooking [3,5,6]. Furthermore, cooking videos have been shown to improve culinary skills and self-efficacy, reinforcing the appeal of engaging in cooking activities [2,7].

Social media algorithms tend to reinforce users’ existing preferences, creating information “bubbles” that restrict exposure to diverse viewpoints [8]. Educational videos disseminated through video-sharing platforms can rapidly reach a broad audience via social media, making them an effective tool for public engagement [9,10,11]. Cooking videos in particular have gained popularity as an alternative to formal culinary education, providing accessible means for acquiring knowledge about health and nutrition [2,12]. Research by Ngqangashe and De Backer [4] indicates that video content influences individuals’ food choices, food preferences, and their intention to consume and prepare the foods presented in the videos. For instance, a study in Israel identified a positive correlation between social media usage and symptoms of disordered eating [13]. Additionally, a nationally representative study conducted by Sidani et al. [14] on young adults aged 19 to 32 in the United States found a correlation between high social media engagement and problematic eating behaviours. Similarly, research by Chung et al. [1] highlights the impact of social media on adolescents’ dietary habits, demonstrating both positive effects, such as increased fruit and vegetable intake, and negative influences, such as unhealthy eating behaviours linked to fast food advertising. This highlights the significant impact that social media algorithms can have on shaping individuals’ dietary habits and eating behaviours.

Therefore, cooking, dietary, and health-related videos disseminated through social media have become increasingly linked to individuals’ health and eating behaviours. Healthy eating is generally defined as consuming more fruits and vegetables, maintaining a balanced diet, and incorporating a variety of foods, while limiting the intake of high-calorie foods and avoiding processed products [15,16]. Furthermore, research has emphasised the necessity for healthcare professionals to utilise social media platforms to disseminate accurate and reliable health information to the general public, thereby enhancing individuals’ knowledge of healthy eating [10,16]. While social media has been recognised as an influential tool for conveying cooking-related information, particularly to younger generations [10,17], there remains a lack of research on public perceptions of online cooking content [18].

Currently, Instagram is one of the most popular social media platforms, serving as an accessible and practical medium for sharing health-related dietary and nutritional information, particularly through video content [19,20]. Additionally, several studies have investigated Instagram users in Taiwan [21,22], highlighting its significance as a key social media platform in the country.

Existing research on cooking videos and dietary health has predominantly focused on students or younger populations [1,14,18,23]. However, there is a notable lack of studies examining these topics among adults, particularly those in established adulthood (aged 30–45) [24,25]. Individuals in this life stage often navigate multiple roles and responsibilities, which can contribute to increased stress levels and decreased well-being [24,25,26]. High stress levels have been associated with an increased consumption of ultra-processed foods and unhealthy dietary behaviours [27,28]. Additionally, research on middle-aged adults has identified a common pattern of unhealthy lifestyle behaviours, with the simultaneous presence of both physical inactivity and poor dietary habits [29]. These findings underscore the need for further investigation into effective strategies for promoting healthier eating habits among adults in established adulthood, particularly through digital platforms such as social media.

### Research Objectives and Hypotheses

The primary objective of this study is to examine whether exposure to healthy cooking videos on Instagram influences healthy eating behaviours among adults in established adulthood in Taiwan. Specifically, this study investigates the relationships between cooking behaviour, engagement with healthy cooking techniques multimedia, and healthy eating behaviours among individuals who regularly engage in home cooking. By focusing on adults who incorporate cooking into their daily routines, this research aims to provide deeper insights into the impact of digital cooking content on dietary choices and to explore potential strategies for leveraging social media as a tool for promoting healthier eating habits within this demographic.

The findings of this study are expected to contribute to the existing body of literature on adult nutrition by elucidating the mechanisms through which digital culinary content influences food-related behaviours. Furthermore, the study aims to serve as a valuable reference for policymakers and public health initiatives, offering empirical evidence to support the development of social media-based interventions aimed at enhancing healthy eating behaviours.

To address the research objectives, the following hypotheses are proposed:

**H1**:  *Cooking behaviour has a significant positive impact on engagement with healthy cooking techniques multimedia.*

**H2**:  *Cooking behaviour has a significant positive impact on healthy eating behaviours.*

**H3**:  *Engagement with healthy cooking techniques multimedia has a significant positive impact on healthy eating behaviours.*

**H4**:  *Engagement with healthy cooking techniques multimedia mediates the relationship between cooking behaviour and healthy eating behaviours.*

## 2. Materials and Methods

### 2.1. Study Design

A cross-sectional online survey was conducted among adults in established adulthood (aged 30–45) in Taiwan who regularly engage in daily cooking to examine whether their use of the social media platform Instagram to watch healthy cooking techniques multimedia influences their healthy eating behaviours. This study strictly adheres to the principles outlined by the National Taiwan University Research Ethics Centre (approval number: 202405ES089) and the Declaration of Helsinki.

### 2.2. Participant Eligibility Criteria

Potential participants were eligible for this study if they met the following criteria: (1) aged between 30 and 45 years, (2) residing in Taiwan, (3) using Instagram to watch cooking tutorials or food preparation videos, and (4) regularly engaging in cooking in daily life, with a minimum of one home-cooked dinner per week. Individuals who did not meet these eligibility criteria were excluded from participation.

### 2.3. Recruitment Methods

CFA/SEM is a large-sample technique [30]. In CFA/SEM analysis, it is commonly recommended to use the ratio of the sample size (N) to the number of free parameters (q) (N:q) as a guiding principle for evaluation. A frequently cited N:q ratio typically ranges between 10:1 and 20:1 [30,31,32]. Therefore, considering the statistical requirements for sample size, the effective questionnaire response rate, and the strict participant screening criteria, this study initially collected 600 questionnaires to validate the model. Ultimately, 488 valid responses were obtained, which still met the predetermined sample size requirements.

Recruitment for this study began on 1 August 2024, and continued until the target sample size of 600 participants was reached. To ensure participant diversity, a combination of convenience sampling and snowball sampling methods was employed. To maintain the integrity of the sample, individuals who did not meet any of the recruitment criteria were systematically excluded. The screening process was implemented at the beginning of the questionnaire, requiring participants to confirm their eligibility before proceeding. During the data cleaning process, any responses that did not meet the inclusion criteria or exhibited inconsistencies—such as not residing in Taiwan or failing to meet the required cooking frequency—were removed. Ultimately, 488 valid questionnaires were collected from the initial pool of respondents, resulting in an effective response rate of 81.3%. This rigorous screening approach ensured that the final dataset accurately represented the study’s target population. Recruitment efforts included posting announcements on social media platforms such as Instagram and Facebook, particularly within cooking-related groups. Additionally, study notices were displayed on bulletin boards in cooking technique training classrooms to attract individuals actively engaged in culinary practices. The recruitment materials provided a clear overview of the study, describing it as an online survey designed to explore the content and influence of cooking technique videos shared on Instagram.

### 2.4. Study Procedure

Individuals interested in participating could join the study by scanning a Quick Response (QR) code or accessing the webpage link provided on the recruitment poster to complete an initial online screening questionnaire. Those who met the eligibility criteria were then provided with a Participant Information Statement and an Informed Consent Form. Upon giving their informed consent, participants proceeded to complete a demographic questionnaire, which gathered information on gender (male, female, prefer not to disclose), age range [30,31,32,33,34,35,36,37,38,39,40,41,42,43,44,45], education level, and current living status. Following the demographic section, participants answered the main research questionnaire, which assessed key constructs, including Cooking Behaviour, Healthy Cooking Techniques Multimedia, and Healthy Eating Behaviours. The survey was designed to explore the relationship between engagement with healthy cooking content on social media and dietary behaviours. Participation was entirely voluntary and participants had the option to withdraw from the study at any time without consequences. To ensure proper compensation for their time, participants were required to provide a valid email address at the end of the survey, through which they would receive a gift card upon study completion.

The recruitment process remained open until the target sample size of 600 participants was reached. After data collection was completed, all submitted email addresses were systematically checked for duplication to prevent multiple submissions from the same participant, ensuring data accuracy and reliability. The study adhered to ethical research guidelines to maintain participant confidentiality and data integrity.

### 2.5. Measurement Tools and Data Analysis

#### 2.5.1. Measurement Tools

The research scale was independently translated by two bilingual researchers proficient in both Chinese and English with relevant professional backgrounds. The initial translation was reviewed by five experts in the fields of hospitality, healthy eating, and psychology, and modifications were made based on their feedback to ensure semantic accuracy and cultural appropriateness. In cases of expert disagreement, the research team first discussed and reached a consensus before seeking feedback from the target participants to ensure clarity and comprehension of the questionnaire.

The Cooking Behaviour questionnaire was developed based on the survey instruments and findings of Pinard et al. [33], Lahne, Wolfson, and Trubek [34], Namin et al. [35], Lins et al. [36], and Jomori et al. [37]. The questionnaire items were further reviewed and validated by five experts to ensure their clarity and relevance. Its primary purpose is to assess participants’ cooking behaviours. Higher scores on these items indicate a greater self-perceived proficiency in cooking behaviour.

The Healthy Cooking Techniques Multimedia questionnaire was developed based on the survey instruments and findings of Camargo et al. [18], Surgenor et al. [12], Nour et al. [7], and Raber et al. [38]. The questionnaire items were further reviewed and validated by five experts to ensure their relevance and clarity. Its primary purpose is to assess participants’ engagement with Healthy Cooking Techniques Multimedia. Higher scores on these items indicate a greater level of involvement with Healthy Cooking Techniques Multimedia.

The Healthy Eating Behaviours questionnaire was developed based on the survey instruments and findings from Guertin, Pelletier, and Pope [39], Żakowska-Biemans et al. [40], and Köksal et al. [41]. The questionnaire items were further reviewed and validated by five experts to ensure their clarity and relevance. It was designed to assess participants’ individual healthy eating behaviours. Higher scores on these items indicate a greater adherence to healthy eating behaviours.

All indicators in the study were measured using a seven-point Likert scale, where 1 indicated “strongly disagree” and 7 indicated “strongly agree”.

#### 2.5.2. Data Analysis

Data analysis was conducted using SPSS 25.0 and AMOS 23.0 software. The analytical procedures included descriptive statistics, correlation analysis, reliability and validity analysis, confirmatory factor analysis (CFA), and structural equation modelling (SEM) to validate the research hypotheses and assess model fit. This comprehensive approach to data analysis enabled a thorough examination of the relationships between variables and the overall suitability of the model.

## 3. Results

### 3.1. Questionnaire and Demographic Variables Analysis

As presented in Table 1, the demographic characteristics of the participants are as follows. Regarding gender distribution, 215 participants (44.1%) identified as male, 262 participants (53.7%) identified as female, and 11 participants (2.3%) preferred not to disclose their gender. In terms of age, the majority of participants were between 30 and 35 years old (*n* = 225, 46.1%), followed by those aged 36 to 40 years (*n* = 146, 29.9%), and those aged 41 to 45 years (*n* = 117, 24.0%). In terms of educational attainment, the largest proportion of participants held a university degree (*n* = 232, 47.5%). Regarding living arrangements, the majority of participants resided with family members (two or more individuals), accounting for 341 participants (69.9%) of the sample.

### 3.2. Descriptive Analysis of Each Construct

This study conducted a mean and standard deviation analysis for cooking behaviour, healthy cooking techniques multimedia, and healthy eating behaviours. A higher mean score indicates a greater level of agreement with the respective construct, as presented in Table A1.

#### 3.2.1. Cooking Behaviour

As shown in Table A1, in the cooking behaviour construct, participants most strongly agreed with the statement, “I am capable of following recipes or instructional videos to prepare new dishes” (M = 4.71). This was followed by “I am confident in accurately measuring ingredient portions and determining appropriate cooking times” (M = 4.64), and “I enjoy cooking because it provides me with relaxation and a sense of enjoyment” (M = 4.57). The overall mean score for the cooking behaviour construct was 4.41.

#### 3.2.2. Healthy Cooking Techniques Multimedia

As shown in Table A1, in the healthy cooking techniques multimedia construct, participants most strongly agreed with the statement, “Through these videos, I have learned healthier cooking methods (such as reducing fat usage and incorporating more natural ingredients)” (M = 5.51). This was followed by “I frequently watch multimedia content about healthy cooking techniques (e.g., Instagram)” (M = 5.47), and “Watching these videos has increased my confidence in preparing healthy meals” (M = 5.26). The overall mean score for the healthy cooking techniques multimedia construct was 5.39.

#### 3.2.3. Healthy Eating Behaviours

As shown in Table A1, in the healthy eating behaviours construct, participants most strongly agreed with the statement, “After watching healthy cooking videos, I make an effort to use healthier ingredients, such as substituting butter with olive oil” (M = 5.41). This was followed by “After watching healthy cooking videos, I pay attention to nutrition labels when purchasing ingredients to select healthier products” (M = 5.24), and “I believe that the knowledge acquired from watching healthy cooking videos will influence my dietary habits for at least one year or longer” (M = 4.72). The overall mean score for the healthy eating behaviours construct was 5.08.

#### 3.2.4. Pearson Correlation Analysis

As shown in Table 2, Pearson correlation analysis was used to explore the correlations between each construct. The correlation coefficient method was employed, with the rule that the correlation value should not exceed 0.85 [30]. The analysis results, as shown in Table 3, indicate that cooking behaviour and healthy cooking techniques multimedia are positively correlated to a moderate degree (r = 0.345, *p* < 0.001); cooking behaviour and healthy eating behaviours are also positively correlated to a moderate degree (r = 0.347, *p* < 0.001); and healthy cooking techniques multimedia and healthy eating behaviours are positively correlated to a moderate degree as well (r = 0.551, *p* < 0.001).

#### 3.2.5. Confirmatory Factor Analysis and Model Fit Testing

In this study, variables were measured using a self-report approach, collecting information from a single respondent. This method may introduce common method variance bias. To examine the potential presence of common method variance, this study employed Harman’s single-factor test [41]. An exploratory factor analysis indicated that the cumulative variance explained was 74.7%, with the first factor accounting for 43.8% of the variance. Since the variance explained by the first factor was below the 50% threshold, common method variance was determined to have a negligible impact.

The model in this study was tested using standards proposed by multiple scholars. The criteria for confirmatory factor analysis (CFA) include a Cronbach’s α and composite reliability (CR) value greater than 0.7, and average variance extracted (AVE) greater than 0.36. For the structural equation modeling (SEM) model fit indices, the standards are as follows: a smaller χ^2^ is better, higher degrees of freedom (df) are preferred, χ^2^/df should be less than 5, goodness of fit index (GFI) and adjusted goodness of fit index (AGFI) values above 0.8 are acceptable, standardised root mean square residual (SRMR) should be less than 0.1, and root mean square error of approximation (RMSEA) should be less than 0.1. Additionally, the comparative fit index (CFI), normed fit index (NFI), and non-normed fit index (NNFI) values should be greater than 0.9 [30,43,44,45,46]. In some cases, NFI may be acceptable with a threshold of 0.8 [47]. As shown in Table 3, all indicators and fit indices meet the acceptable criteria, indicating that the model can effectively explain the relationships between the constructs.

### 3.3. Structural Equation Modeling Analysis

**Figure 1:**
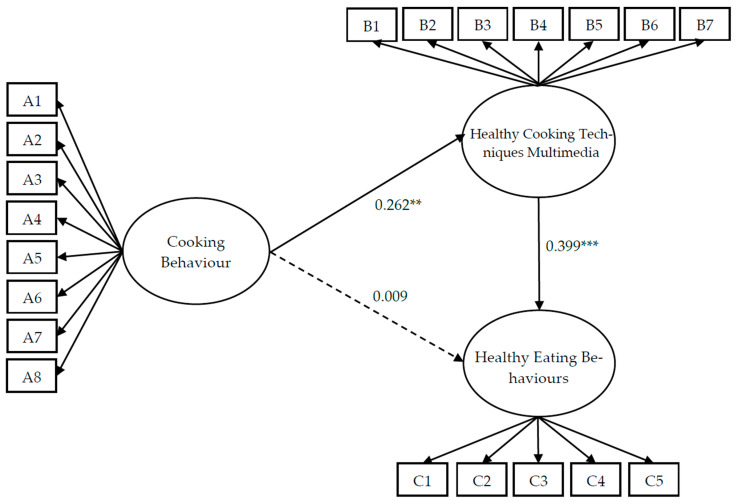
Structural equation model (SEM) illustrating the mediating role of healthy cooking techniques multimedia in the relationship between cooking behaviour and healthy eating behaviours. Latent variables are represented by ellipses and observed indicators by rectangles. All paths are standardised and statistically significant (*p* < 0.05). Note: ** *p* < 0.01, *** *p* < 0.001.

The results in Figure 1 and Table 4 show that cooking behaviour has a significant positive impact on healthy cooking techniques multimedia, with a path coefficient of 0.262 **. Therefore, H1 is empirically supported. However, cooking behaviour does not have a significant positive impact on healthy eating behaviours, as indicated by a path coefficient of 0.009; thus, H2 is not supported. Meanwhile, healthy cooking techniques multimedia has a significant positive impact on healthy eating behaviours, with a path coefficient of 0.399 ***, confirming support for H3.

The bootstrap method was used to determine whether the (1 − α)% confidence interval includes zero. If zero is not included, it indicates statistical significance at the α level. Following the recommendations of Hayes [48] and MacKinnon and Fairchild [49], a 95% confidence interval and 2000 bootstrap samples were used. The results show that healthy cooking techniques multimedia mediates the relationship between cooking behaviour and healthy eating behaviours, as neither the direct nor the indirect effects include zero. This indicates that healthy cooking techniques multimedia has a partial mediating effect, thus supporting H4.

## 4. Discussion

This study examines whether the healthy eating behaviours of adults in established adulthood (aged 30 to 45) in Taiwan are influenced by their cooking behaviour and exposure to healthy cooking techniques videos, particularly those shared on the social media platform Instagram. The research was conducted by selecting appropriate participants and focusing on individuals aged 30 to 45, a life stage characterised by multiple pressures from family, work, and daily life. Excessive stress during this period has been linked to unhealthy dietary habits. The findings of this study indicate that engaging in cooking as part of daily life does not directly influence healthy eating behaviours. However, watching healthy cooking techniques videos can significantly impact adults’ dietary habits, suggesting that social media-based cooking content may serve as an effective tool for promoting healthier eating behaviours among this demographic.

Although there is a certain association between cooking behaviour and healthy eating, simply engaging in cooking does not necessarily promote healthy eating, as food choices and cooking methods are still influenced by existing dietary habits, nutritional knowledge, and environmental factors [50]. In contrast, engaging with digital content, particularly watching healthy cooking videos, can enhance self-efficacy through visual demonstrations and observational learning, making individuals more confident in selecting healthier ingredients and cooking methods [51,52,53]. Furthermore, digital content provides continuous reinforcement through real-time feedback, interactive learning, and social support, increasing individuals’ motivation and commitment to healthy eating [54]. Therefore, compared to cooking alone, digital content plays a more active role in behaviour change, further fostering the development of healthy eating habits. Additionally, individuals who prioritise healthy eating are more likely to actively seek out healthy cooking videos or, due to algorithmic filtering, be exposed to a more limited range of video content, suggesting that behaviour change may not be solely driven by the videos themselves [13,55,56].

**Figure 2:**
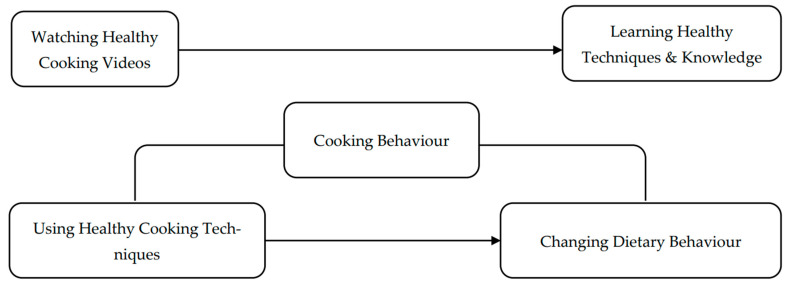
Conceptual model showing how adopting healthy cooking techniques mediates the relationship between cooking behaviour and dietary behaviour change.

As summarised [51,52,53] in Figure 2, our proposed model illustrates the behavioural pathway revealed by the empirical results, highlighting the mediating role of adopting healthy cooking techniques in linking general cooking behaviour to healthier dietary outcomes. While cooking behaviour alone did not directly predict improved eating habits, our findings suggest that individuals are more likely to change their dietary patterns when they actively apply healthy cooking techniques––an effect that is further supported by learning from visual and interactive cooking content, particularly on social media platforms such as Instagram. This process aligns with social cognitive theory, wherein observational learning enhances cooking self-efficacy and nutritional awareness. Although media engagement and learning motivation are not explicitly depicted in the diagram, they are conceptually represented by the acquisition of healthy techniques and knowledge. Therefore, Figure 2 conceptually captures this simplified pathway, illustrating how the practical application of acquired knowledge fosters positive changes in dietary behaviour.

Cooking content on Instagram is often scripted from a perspective that emphasises healthy nutrition or sharing cooking techniques [57,58,59]. Through watching cooking videos, individuals can gradually internalise knowledge, whether by improving their culinary skills or enhancing their understanding of nutrition and healthy eating [4,7]. Instagram serves as an effective platform for disseminating and sharing this information through video content. These findings align with previous research, which highlights that digital media—particularly video-based platforms—are powerful tools for health communication and behavioural change [13,19,20,50].

A key finding of this study is that healthy cooking videos can influence individuals’ healthy eating behaviours. Those who cook regularly and actively engage with cooking-related content on Instagram are more likely to adopt healthier dietary habits [2,12]. However, social media algorithms may also contribute to issues related to disordered eating [13]. This suggests that the visual and interactive nature of social media plays a significant role in shaping health-related attitudes and behaviours. These findings align with a growing body of literature emphasizing the effectiveness of social media in promoting health and nutrition education.

From a theoretical perspective, this study contributes to the understanding of how digital media influences healthy behaviours, particularly within the framework of social cognitive theory [23,50,51]. The role of observational learning in shaping dietary habits is evident, as individuals exposed to healthy cooking demonstrations are more likely to adopt these techniques in their daily lives. Furthermore, this study highlights the importance of media engagement in facilitating behavioural change, demonstrating that platforms like Instagram can serve as effective channels for health promotion. These findings are consistent with the research of Chan & Allman-Farinelli [19], Sbardelotto, Martins, & Buss [20].

From a practical perspective, the study’s findings hold significant implications for public health initiatives and content creators. Health organizations and educators can leverage social media platforms to disseminate evidence-based cooking and nutrition content targeted at specific demographic groups [10,16]. Additionally, culinary influencers and health advocates can enhance their impact by incorporating interactive elements, such as live demonstrations, step-by-step tutorials, and user-generated content, to further engage audiences and facilitate learning.

Overall, this study focuses on examining the relationship between cooking behaviour, healthy cooking techniques multimedia, and healthy eating behaviours among adults in established adulthood (aged 30–45)—a demographic that has received limited attention in previous research on social media and healthy eating behaviours. By addressing this gap, the study makes a meaningful contribution to the field by expanding knowledge on how digital cooking content can influence dietary practices in this population.

## 5. Research Limitations and Future Directions

This study offers important insights into the relationship between healthy cooking videos on Instagram and healthy eating behaviours among adults in established adulthood. Nevertheless, some aspects should be taken into consideration when interpreting the findings. The participant sample primarily consisted of individuals with higher educational attainment and those within the younger range of established adulthood, which may influence the generalizability of the results. Future studies could benefit from including more diverse demographic groups to examine whether these relationships hold across different backgrounds and life stages.

The study design did not account for participants’ level of health awareness, which may influence their engagement with health-related content. Individuals who are more health-conscious might seek out such content due to their existing awareness or be targeted by social media algorithms rather than being directly influenced by the video content itself. Future research should consider assessing participants’ health consciousness and employ a stratified analysis approach to better examine the relationship between social media exposure and healthy dietary choices.

Furthermore, the data were based on self-reported measures, which may be influenced by individual perceptions and recall. Future research might strengthen these findings by incorporating complementary methods, such as detailed dietary assessments, observational approaches, or digital analysis of social media engagement.

This study focused specifically on Instagram as the platform of interest. Given the growing role of multiple social media platforms in disseminating cooking-related content, such as YouTube and TikTok, future investigations could explore whether similar effects on healthy eating behaviours are observed across different platforms with varying formats and modes of user interaction.

In addition, exploring a wider range of health-related outcomes—such as improvements in nutritional knowledge, cooking self-efficacy, and psychological well-being—would contribute to a more comprehensive understanding of how engagement with healthy cooking content may support overall health.

By addressing these considerations, future research can expand upon the present study’s contributions and further clarify the potential of social media as a tool for promoting healthier eating practices among adults in established adulthood.

## 6. Conclusions

This study surveyed adults aged 30 to 45 in Taiwan to examine whether watching healthy cooking techniques videos on Instagram influences the healthy eating behaviours of individuals who cook in their daily lives. Accordingly, the study explores this relationship through the constructs of cooking behaviour, Healthy Cooking Techniques Multimedia, and Healthy Eating Behaviours. Overall, in this sample of adults aged 30 to 45 in Taiwan, healthy cooking techniques videos shared on Instagram have the potential to influence viewers’ healthy eating behaviours. This effect is particularly evident among individuals who cook regularly and engage with cooking-related content on social media, as the health knowledge and cooking techniques presented in these videos can contribute to positive dietary changes.

These findings have important public health implications, particularly in digital health promotion. Given the widespread use of social media, platforms like Instagram can serve as effective tools for promoting nutrition education and healthier cooking practices. Health organizations and policymakers could leverage social media to develop targeted campaigns that incorporate engaging cooking tutorials and evidence-based dietary guidance. Additionally, collaborations with culinary influencers and nutrition experts could enhance the credibility and reach of such content, encouraging broader adoption of healthy cooking habits. By utilising social media’s interactive and visual nature, public health initiatives can effectively promote sustainable dietary improvements and contribute to long-term population health.

## Figures and Tables

**Table 1:** Participant characteristics (*n* = 488).

Background Variable	Category	Frequency	Percentage (%)
Gender	Male	215	44.1
	Female	262	53.7
	Prefer not to disclose	11	2.3
Age	30–35 years old	225	46.1
	36–40 years old	146	29.9
	41–45 years old	117	24.0
Educational background	Elementary school or below	6	1.2
	Junior high school (middle school)	69	14.1
	High school or vocational school	55	11.3
	Five-year junior college	126	25.8
	University	232	47.5
	Graduate school or above	6	1.2
Living situation	Living alone	51	10.5
	Living with spouse only (2 people)	84	17.2
	Living with family (more than 2 people)	341	69.9
	Other	12	2.5

**Table 2:** Correlation Analysis of Dimensions.

Dimension	Cooking Behaviour	Healthy Cooking Techniques Multimedia	Healthy Eating Behaviours
Cooking Behaviour	1		
Healthy Cooking Techniques Multimedia	0.345 **	1	
Healthy Eating Behaviours	0.347 **	0.551 **	1

** *p* < 0.001.

**Table 3:** Confirmatory Factor Analysis.

Dimension	Item Description				Mean	Standardised Factor Loading	Cronbach’s α	CR	AVE
Cooking Behaviour	I frequently prepare my own meals rather than eating out.				4.54	0.893	0.956	0.956	0.732
	I am capable of following recipes or instructional videos to prepare new dishes.				4.71	0.905			
	I have the ability to use various cooking techniques (such as pan-frying, stir-frying, baking, and stewing).				4.28	0.913			
	I regularly plan my weekly meals and prepare ingredients in advance.				4.48	0.941			
	I consider myself proficient in cooking a variety of basic dishes.				4.44	0.871			
	I am confident in accurately measuring ingredient portions and determining appropriate cooking times.				4.64	0.803			
	I enjoy cooking because it provides me with relaxation and a sense of enjoyment.				4.57	0.73			
	My primary motivation for cooking is to maintain a healthy diet.				3.60	0.762			
Healthy Cooking Techniques Multimedia	I frequently watch multimedia content about healthy cooking techniques (e.g., Instagram).				5.47	0.788	0.932	0.933	0.667
	I watch videos or programs related to healthy cooking at least once a week.				5.29	0.832			
	Compared to general cooking programs, I prefer watching videos that feature healthy cooking techniques.				5.39	0.894			
	After watching healthy cooking videos, I try to apply the techniques I have learned in my own cooking.				5.46	0.866			
	Watching these videos has increased my confidence in preparing healthy meals.				5.26	0.858			
	I adjust my dietary choices based on the recommendations provided in these videos.				5.34	0.702			
	Through these videos, I have learned healthier cooking methods (such as reducing fat usage and incorporating more natural ingredients).				5.51	0.759			
Healthy Eating Behaviours	I believe that the knowledge acquired from watching healthy cooking videos will influence my dietary habits for at least one year or longer.				4.72	0.724	0.917	0.917	0.691
	After watching healthy cooking videos, I consciously reduce the use of high-fat, high-salt, and high-sugar cooking methods.				4.81	0.699			
	After watching healthy cooking videos, I make an effort to use healthier ingredients, such as substituting butter with olive oil.				5.41	0.897			
	After watching healthy cooking videos, I pay attention to nutrition labels when purchasing ingredients to select healthier products.				5.24	0.94			
	I modify my cooking methods based on recommendations from healthy cooking videos to make meals healthier.				5.21	0.867			
CFA results	χ^2^	df	CFI	RMSEA	SRMR	NFI	NNFI	GFI	AGFI
	1365.236	334	0.912	0.064	0.058	0.887	0.900	0.844	0.804

Note: *n* = 488. CR = Composite Reliability; AVE = Average Variance Extracted; CFI = Bentler’s Comparative Fit Index; RMSEA = Root Mean Square Error of Approximation; SRMR = Standardised Root Mean Square Residual; NFI = Normed Fit Index; NNFI = Non-Normed Fit Index; GFI = Goodness-of-Fit Index; AGFI = Adjusted Goodness-of-Fit Index.

**Table 4:** SEM Analysis Results and Mediation Effect Test of Cooking Behaviour Influence on Healthy Eating Behaviours through Healthy Cooking Techniques Multimedia.

Parameter	Estimate	Bootstrapping				Hypothesis	Result
		Bias-Corrected 95% CI		Percentile 95% CI			
		Lower	Upper	Lower	Upper		
Indirect Effect							
Cooking Behaviour -> Healthy Cooking Techniques Multimedia -> Healthy Eating Behaviours	0.105 *	0.044	0.204	0.038	0.185	H4	Supported
Direct Effect							
Cooking Behaviour -> Healthy Cooking Techniques Multimedia	0.262 **	0.097	0.470	0.094	0.465	H1	Supported
Cooking Behaviour -> Healthy Eating Behaviours	0.009	−0.158	0.158	−0.140	0.166	H2	Not Supported
Healthy Cooking Techniques Multimedia -> Healthy Eating Behaviours	0.399 ***	0.248	0.575	0.246	0.573	H3	Supported
Overall Effect							
Cooking Behaviour -> Healthy Eating Behaviours	0.114	−0.048	0.275	−0.042	0.280		

Note: * *p* < 0.05, ** *p* < 0.01, *** *p* < 0.001.

## Data Availability

Due to research ethics considerations, the data cannot be made available.

## Appendix A

**Table A1:** Descriptive Analysis of Each Construct.

Dimension	Item Description	Mean	Standard Deviation	95% CI for the Difference	
				Lower Bound	Upper Bound
Cooking Behaviour	I frequently prepare my own meals rather than eating out.	4.54	1.706	4.30	4.52
	I am capable of following recipes or instructional videos to prepare new dishes.	4.71	1.747		
	I have the ability to use various cooking techniques (such as pan-frying, stir-frying, baking, and stewing).	4.28	1.764		
	I regularly plan my weekly meals and prepare ingredients in advance.	4.48	1.769		
	I consider myself proficient in cooking a variety of basic dishes.	4.44	1.798		
	I am confident in accurately measuring ingredient portions and determining appropriate cooking times.	4.64	1.747		
	I enjoy cooking because it provides me with relaxation and a sense of enjoyment.	4.57	1.714		
	My primary motivation for cooking is to maintain a healthy diet.	3.60	1.792		
	Overall Mean	4.41	1.755		
Healthy Cooking Techniques Multimedia	I frequently watch multimedia content about healthy cooking techniques (e.g., Instagram).	5.47	1.266	5.32	5.48
	I watch videos or programs related to healthy cooking at least once a week.	5.29	1.282		
	Compared to general cooking programs, I prefer watching videos that feature healthy cooking techniques.	5.39	1.291		
	After watching healthy cooking videos, I try to apply the techniques I have learned in my own cooking.	5.46	1.277		
	Watching these videos has increased my confidence in preparing healthy meals.	5.26	1.366		
	I adjust my dietary choices based on the recommendations provided in these videos.	5.34	1.358		
	Through these videos, I have learned healthier cooking methods (such as reducing fat usage and incorporating more natural ingredients).	5.51	1.371		
	Overall Mean	5.39	1.316		
Healthy Eating Behaviours	I believe that the knowledge acquired from watching healthy cooking videos will influence my dietary habits for at least one year or longer.	4.72	1.397	4.93	5.09
	After watching healthy cooking videos, I consciously reduce the use of high-fat, high-salt, and high-sugar cooking methods.	4.81	1.356		
	After watching healthy cooking videos, I make an effort to use healthier ingredients, such as substituting butter with olive oil.	5.41	1.333		
	After watching healthy cooking videos, I pay attention to nutrition labels when purchasing ingredients to select healthier products.	5.24	1.383		
	I modify my cooking methods based on recommendations from healthy cooking videos to make meals healthier.	5.21	1.394		
	Overall Mean	5.08	1.373		
